# Estimation of the value-based price of a blood test for Alzheimer’s disease pathology in primary and specialty care in the U.S.

**DOI:** 10.1016/j.tjpad.2025.100219

**Published:** 2025-06-09

**Authors:** Soeren Mattke, Jiahe Chen, Mark Hanson, Kim G. Johnson, Cara Leahy, David A. Merrill, Victoria Shada, Jorge G. Ruiz

**Affiliations:** a The USC Brain Health Observatory, USC Dornsife, Los Angeles, CA 90089, USA; b Department of Neurology, Duke University School of Medicine, Durham, NC 27514, USA; c Memorial Healthcare Institute for Neuroscience, Owosso, MI 48867, USA; d Pacific Brain Health Center, Pacific Neuroscience Institute, Santa Monica, CA 90404, USA; e Department of Internal Medicine, Section on Gerontology and Geriatric Medicine, Wake Forest School of Medicine, Winston-Salem, NC 27157, USA; f Memorial Healthcare System, Hollywood, FL 33021, USA

**Keywords:** Alzheimer’s disease, Blood biomarker, Economic value, Cost-effectiveness

## Abstract

**Background:**

Blood tests for the pathology of Alzheimer’s disease (AD) are emerging as alternative to amyloid PET scans and analysis of cerebrospinal fluid. (CSF). However, their economic value, which depends on test accuracy as well as effect on clinical decision-making, remains unclear.

**Methods:**

We use a Markov model to estimate the value-based price of a blood test with sensitivity of 88 % and specificity of 89 %, if labeled for triage and confirmation of the AD pathology in primary and specialty care. The value-based price was defined as price of the test, at which overall diagnostic cost per true positive case of early-stage AD would equate that under standard of care (identification in primary care and referral to specialty care based on the results of a brief cognitive test). Assumptions for the effect of test use on clinical decisions came from a structured expert consultation process.

**Results:**

If used in primary care, the value-based price would be $290 for a triage and $1150 for a confirmatory test, respectively, as use of PET or CSF testing would decline by 47 % and 86 %, respectively. If used in specialty care, i.e., after confirmation of early-stage cognitive impairment, the overall number of blood tests would decline. Consequently, the value-based price would increase to $450 for a triage test and $1950 for a confirmatory test.

**Conclusions:**

The results project substantial cost savings from implementing a blood test for AD pathology within the diagnostic pathway based on modeling results, which future research should confirm with actual data.

## 1 Introduction

The emergence of disease-modifying treatments for Alzheimer’s disease (AD), as the first option to alter the trajectory of the disease, has attracted substantial attention to the treatment eligibility process. Based on current FDA labeling, to potentially qualify, patients require clinical confirmation of early-stage cognitive impairment, i.e., mild cognitive impairment (MCI) or mild dementia, and determination of AD pathology - indicated by the presence of brain amyloid. In addition, the decision to treat requires careful weighing of net clinical benefit, contraindications, treatment burden and side effects in this complex, older population. In light of the large size of the potentially treatment-eligible population, several studies have raised concerns about the preparedness of health systems to accommodate the expected demand for diagnostic services [[1], [2], [3]].

One potential bottleneck in the clinical evaluation and care journey for patients is the need to confirm AD pathology, which is conventionally performed with analysis of cerebrospinal fluid (CSF) or positron emission tomography (PET) for the presence of amyloid deposits in the brain. Neither approach is perfectly scalable or accessible: The high fixed cost of a PET scanner means that existing devices are typically run at capacity, adding additional ones is time-consuming and costly, and access in less populated areas is limited. CSF utilization as alternative varies by country, spanning from 5 % in South Korea [4] up to 90 % in Sweden [5], and there are limits to the acceptability of lumbar punctures by patients and also clinicians because of its invasive nature as well as contraindications.

Blood tests for AD pathology have emerged as an attractive solution. Initially conceived as a triage or “rule-out” test to inform the need for confirmatory testing with CSF or PET, their improving accuracy that approaches that of CSF analysis suggests a role as confirmatory or “rule-in” test as well [6]. For example, a recent study that prospectively evaluated the diagnostic accuracy of a blood test that combines the percentage of phosphorylated tau 217 (p-tau217) over non-phosphorylated tau 217 (np-tau217) and the amyloid-β 42/ 40 ratio, referred to as the Precivity AD2 test, showed results comparable to those of CSF analysis [7].

In light of their potentially prominent role in identifying patients with AD pathology, the clinical and economic value of the AD blood tests becomes an important question. Nguyen et al. [8] compared several diagnostic scenarios to determine eligibility for amyloid-targeting treatments in the appropriately indicated patients to the standard of care, i.e., no treatment, and found a pathway containing AD blood test leading to therapy in a subset of the evaluated patients to be not cost-effective. Aye et al. [9] found that using an AD blood test for triage in primary care increased referral rates to specialty care and confirmation of AD diagnosis, but failed to achieve cost-effectiveness under prevailing prices of the subsequent treatment. However, these studies analyzed the cost-effectiveness of a blood test together with the subsequent treatment using amyloid-targeting drugs. This approach seems flawed because it means that the cost-effectiveness of the test becomes a function of the cost-effectiveness of the treatment, whereas the analysis should capture the intrinsic value of the test.

In addition, the value of a test not only depends on its technical properties, such as sensitivity and specificity, but also on how clinicians use the test to inform decisions, i.e., its clinical utility. Both aforementioned studies and also a third publication [10], which projected that use of an AD blood test for triage would reduce cost per diagnosed patient and patient throughput under constrained capacity for AD specialist visits, failed to incorporate this element into the analysis. In this study, we improve upon these limitations and estimate the intrinsic value of a high-performing blood test for AD pathology by estimating the maximum price per test, at which its insertion into the diagnostic process would be cost neutral compared to standard of care, given assumptions for its clinical utility. We analyze use of the test in primary and specialty care settings as well as for triage and confirmation use applications.

## 2 Methods

### 2.1 Overview

We use a Markov model to compare the cost per true positive case of early-stage AD under standard of care (identification in primary care and referral to specialty care based on the results of a brief cognitive test), assuming use of an AD blood test labeled as a “rule out” or “rule in” test. Assumptions for the effect of using the blood test on clinical decisions were obtained with a structured expert consultation process. We model the 10-year timeframe from 2024 to 2033, with use in the U.S. population aged 65 and older.

### 2.2 Model description

The Markov model simulates the journey of patients seeking evaluation for subjective memory complaints or as part of a preventive exam in primary care. It reflects a - highly stylized - patient’s journey through different evaluation stages: the standard of care evaluation is comprised of an initial evaluation by a primary care physician with a brief cognitive assessment, a comprehensive assessment by an AD specialist, and, upon confirmation of early-stage cognitive impairment, confirmatory biomarker testing with positron emission tomography (PET) scanning or cerebrospinal fluid (CSF) testing, leading to a second specialist visit to devise a treatment plan.

The model captures four alternative scenarios that insert a blood test for AD pathology into this diagnostic process. The first two reflect use of the blood test in primary care after suspected early-stage cognitive impairment was detected with a brief cognitive assessment, one as a triage or “rule-out” test and one as a confirmatory or “rule-in” test. The second two scenarios analyze the same two use cases in specialty settings (neurology, geriatric psychiatry, and geriatrics), after early-stage cognitive impairment has been confirmed with a comprehensive assessment. The model has been described in detail in earlier publications [1,10], and the model input parameters and their sources are documented in the Appendix.

### 2.3 Model parameters

#### 2.3.1 Population and disease burden

Data on the U.S. population as well as growth trends and mortality rates were obtained from U.S. Census data. Data for incidence and prevalence of MCI by age group were obtained from prior studies by Gillis et al. [11] and Petersen et al. [12], respectively, and corresponding data for mild dementia from Gillis et al. [13]. The proportion of individuals with early stage cognitive impairment with AD as the underlying pathology by age came from Gustavsson et al. [14]

#### 2.3.2 Blood test

We assume the published performance characteristics of the PrecivityAD2 blood test of a sensitivity of 88 % and a specificity of 89 % for this study [15]. As previously mentioned, the Precivity AD2 blood test measures the p-tau217/np-tau217 ratio and the amyloid-β 42/ 40 ratio to detect amyloid PET positivity.

#### 2.3.3 Expert panel consultation process to derive clinical utility estimates

Data on how AD blood test results inform actual clinical decisions in real-world practice are sparse [16]; therefore we conducted a structured expert consultation process to generate estimates. The expert group included five members, one geriatric psychiatrist, two neurologists, and two geriatricians. We used a modified Delphi approach [17] to obtain their input, in which they received a briefing on the study’s approach and were then asked to provide estimates individually. We instructed the expert to assume that all tests had regulatory approval and coverage, and that clinicians had full autonomy to make decisions based on test results.

**Fig. 1:**
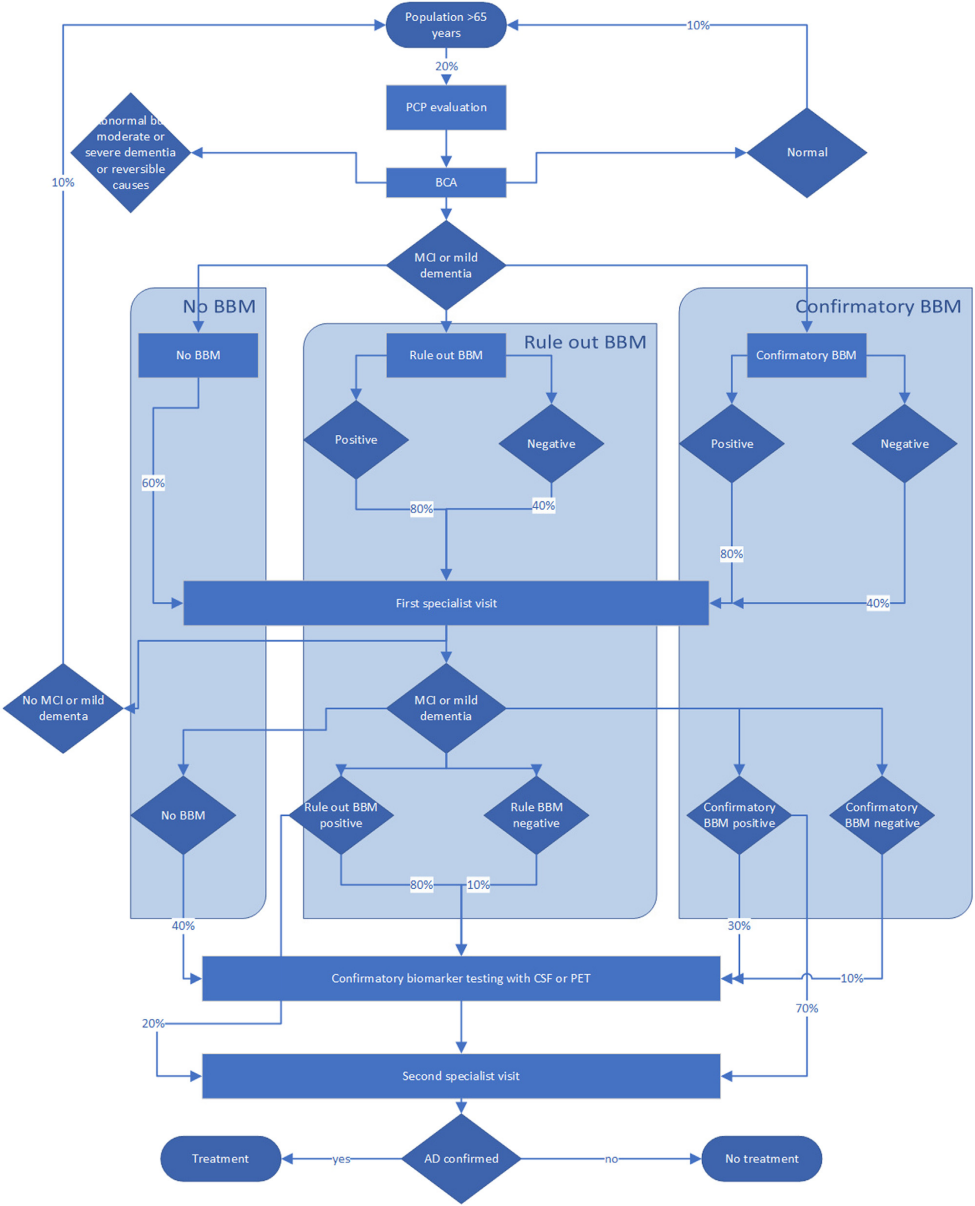
Expert assumptions for diagnostic pathway towards determination of early-stage Alzheimer’s disease.

The answers were compiled and analyzed, and results reported back to the group together with feedback and clarifications, if requested. Subsequently, the experts were given an opportunity to change their estimates, if they considered it appropriate. The median of the final ratings was considered the consensus estimate. Fig. 1 displays those estimates.

The insertion of a blood test is projected to change referral and decision patterns in both primary and specialty care. In the absence of a blood test, 60 % of patients with an abnormal brief cognitive test in primary care would be referred to specialty care. If a blood test were subsequently administered prior to the referral decision, 80 % of those with a positive test and 40 % with a negative result would be referred. Specialists would conduct CSF or PET testing in 40 % of patients with confirmed early-stage cognitive impairment, if no blood test results were available, and in 80 % and 10 % of those with a positive and negative test, respectively, if the blood test were labeled as triage test. If the test were labeled as confirmatory, 30 % and 10 % of cases would undergo additional testing with PET or CSF. In other words, specialists would consider a positive blood test result as sufficient to establish the AD pathology in 20 % of cases, if the test were labeled as triage test, and 70 %, if it were labeled as confirmatory test. The final estimate was that 75 % of confirmatory tests would be based on amyloid PET scans and 25 % on CSF analysis. The diagnostic pathways under those assumptions are illustrated in Fig. 1.

### 2.4 Analysis

We modeled the 10-year timeframe from 2025 to 2034, and used the U.S. population aged 65 and older as the intended use population. This population-level model added the number of individuals, who have aged into the included range and those who previously tested negative during the evaluation process each year. Individuals move through the diagnostic journey towards establishing a definite diagnosis of MCI or mild dementia due to AD as described in Fig. 1 without wait times. Utilization of medical services during this hypothetical journey was tracked and converted to cost based on 2024 Medicare billing rates with a 3 % annual inflation of cost for subsequent years. The rates and billing codes are depicted in Table 1.

**Table 1:** 2024 Medicare rates for services included in model.

Medical service	Billing code	Medicare rate
Primary care office visit	99212	$61.82
Specialist consultation	99204	$182.56
Brain MRI no contrast	70551	$220.02
Lumbar puncture	62270	$159.65
CSF assay for AD pathology	0358U	$312.31
Brain PET Scan	78814	$1480.34
Amyloid PET tracer	A9586	$3257.44

Note: Numeric codes refer to Current Procedure Terminology (CPT) codes and alphanumeric codes to Healthcare Common Procedure Coding System (HCPCS) codes.

The analysis calculated the total cost per confirmed case of early-stage cognitive impairment due to AD under standard of care, i.e., without availability of a blood test for AD pathology. We then estimated cost under the four alternative scenarios described above and derived the price, at which use of a blood test in the diagnostic pathway would be cost-neutral, as the value-based price.

## 3 Results

### 3.1 Use of blood test in primary care

**Fig. 2:**
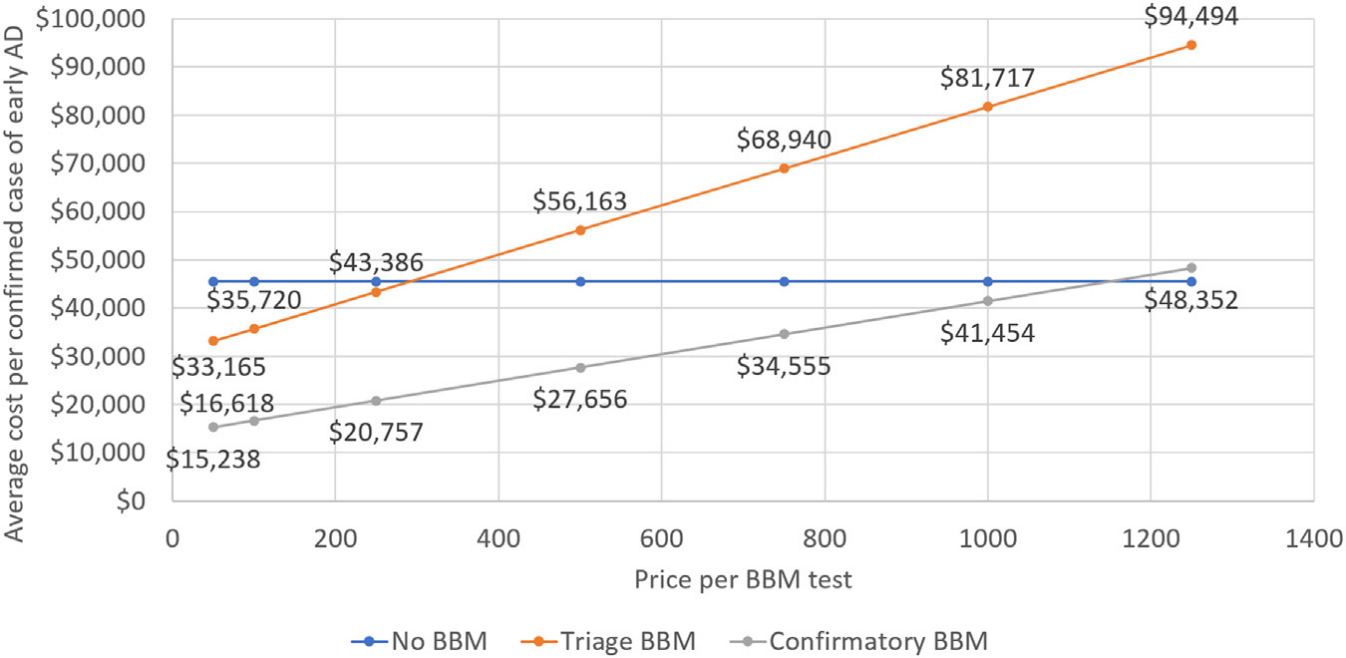
Value-based price for AD blood test used in primary care, assuming labeling as triage and confirmatory test.

The overall medical cost from cognitive screening in primary care to biomarker-confirmed diagnosis is $45,531 per confirmed case of early-stage AD under standard of care, i.e., without use of a blood test. For each confirmed case, 4.7 statistical patients would have undergone confirmatory testing with PET or CSF. Inserting a “rule out” or triage blood test or a “rule in” or confirmatory blood test would reduce that number to 2.5 and 0.7 for a relative reduction of 47 % and 86 %, respectively. Fig. 2 illustrates how the cost of the blood test would influence the cost per confirmed case. The reduction in confirmatory tests means that the price per test, at which it would be cost-neutral – the value-based maximum price -, would be $290 for a triage and $1150 for a confirmatory test, respectively, in the primary care setting in 2025. Referrals to specialty care are projected to decline by 19 % and subsequent specialist visits after completion of the diagnostic journey by 33 %.

A sensitivity analysis explored the effect of test accuracy on estimates of the value-based price. Varying specificity between 85 % and 93 % gave a range from $220 to $360 for the triage test, and from $925 to $1350 for the confirmatory test. Varying sensitivity between 82 % and 92 %, resulted in estimates between $240 and $330 for the triage and between 1050 and $1200 for the confirmatory test.

We explored the effect of using a hypothetical cognitive assessment in primary care with the same accuracy as the blood test of a sensitivity of 88 % and a specificity of 89 %. The cost per accurately diagnosed patient would decline to $39,962 (12 %) under standard of care because of a combination of 28 % fewer specialist visits and 4 % more diagnosed cases. The value-based price for a blood test labeled for triage would increase to $400 and to $1550 for a confirmatory test.

### 3.2 Use of blood test in specialty care

**Fig. 3:**
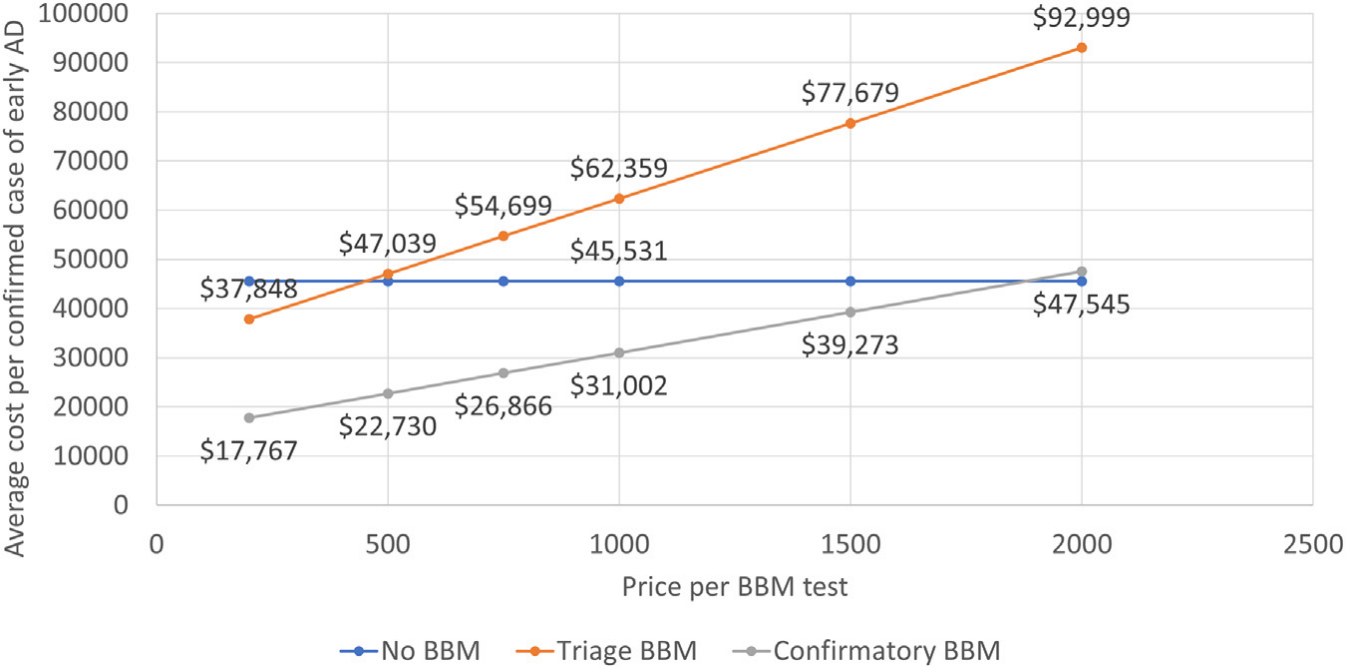
Value-based price for AD blood test used in specialty care, assuming labeling as triage and confirmatory test.

We estimated the effect of the blood test if placed in specialty care as part of the evaluation after referral from primary care. The test would be used **after** confirmation of early-stage cognitive impairment. As this diagnostic step would identify a large proportion of individuals with false positive results on a brief cognitive assessment in primary care, the overall number of blood tests would decline. Consequently, the value-based price would increase to $450 for a triage test and $1950 for a confirmatory test in the specialty care setting (Fig. 3). The estimated relative reduction of use of PET scan and CSF analyses would equal that reported for a blood test placed into primary care settings, because the assumptions for clinician behavior regarding confirmatory testing would not change. Referrals to specialty care would not change but the number of blood tests would be 33 % lower than under standard of care.

Varying specificity between 85 % and 93 % gave a range from $325 to $600 for the triage test, and from $1500 to $2250 for the confirmatory test. Varying sensitivity between 82 % and 92 % resulted in estimates between $350 and $500 for the triage and between $1750 and $2000 for the confirmatory test.

## 4 Discussion

We estimated the effect of inserting a blood test for AD pathology on the cost of the overall diagnostic pathway and projected cost reductions of between $290 and $1950 per case of confirmed early-stage AD depending on the context of use. Put differently, these amounts would represent the maximum value-based prices for such a test. Even the lowest value-based price of $230 for a triage test used in primary care would be considerably higher than the $17 price that CMS had proposed in its draft 2025 rule for an immunoassay. A confirmatory test used in primary care could cost as much as $1150 per test to remain cost neutral, also higher than the proposed $750 price for a mass spectrometry assay. Placing the test into specialty care would increase the maximum value-based prices, as tests would only be conducted in the smaller number of referred patients and after confirmation of early-stage cognitive impairment. However, not using the blood test to inform referral decisions in primary care would remove the expected effect of reducing demand for specialist visits, which are a critical bottleneck in the diagnostic pathway.

Our results differ from those reported by Aye et al., who found a similar pattern of higher value of the blood test when used in specialty care than in primary care, but also that the blood test would increase cost by Euro 603 per case in primary care and decrease it by Euro 178 in specialty care. Similarly, Nguyen et al. stated that triage based on a blood test would increase cost and reduce QALY gains compared to standard of care. However, neither study accounted for a potential behavioral effect of blood test results on referral and testing decisions. Recently published results of the QUIP II study involving the PrecivityAD2 blood test suggest that those behavioral effects could be substantial and therefore need to be incorporated into such projections. Monane et al. provided blood test results for 203 patients of 12 memory clinics and surveyed physicians on how test results influenced their decisions to offer confirmatory testing for AD pathology [16]. A negative test results was associated with a 70 % decline in use of PET or CSF, and a positive result with a 26 % decrease. The overall reduction of 50 % was slightly lower than our estimates of 47 % for a triage and 86 % for a confirmatory blood test. One potential reason is that we instructed our panelists to base their estimates on clinical judgement alone and disregard payer requirements, whereas U.S. payers to date commonly require PET scan or CSF analyses to authorize prescriptions for amyloid-targeting treatments. Indeed, the vast majority (88 %) of clinicians who participated in the QUIP II study agreed that blood test results would be sufficient to establish AD pathology without further testing.

An important finding is that the value-based price for the blood test would be 104 % higher, if used in specialty rather than primary care, because testing only individuals with specialist confirmation of early-stage cognitive impairment would lower the number of blood tests. However, this context of use has the distinct disadvantage of removing the ability of a blood test to inform triage decisions in primary care, thereby reducing the number of specialty referrals. Since specialist appointments are the most salient and also the most difficult to address bottleneck in the diagnostic journey towards determination of treatment eligibility, forgoing this opportunity could increase wait times substantially [18]. For example, a prior modeling study projected that triage in primary care using blood tests for the AD pathology could reduce wait times for specialist appointments by as much as 75 % [1].

A cognitive assessment with higher accuracy would increase the value-based price of the blood test by 34 %, and simultaneously reduce the number of required specialist visits, because fewer cases with a false positive result of early-stage cognitive decline would be referred. Several digital cognitive assessments with potentially higher accuracy that are suitable for primary care are currently in development.

### 4.1 Limitations

Our results should be viewed in the context of the limitations of the study. Model-based results do not constitute directly observed evidence and need to be confirmed with actual data from real-world studies. Similarly, our assumptions for the behavioral effects of a blood test are based on expert input and need to be confirmed. The estimates reflect an assumption that 75 % of confirmatory tests would be based on PET scans, which is probably realistic for the U.S. at this point in time, but may be lower in the future and in other jurisdictions. Increasing the share of CSF analysis would decrease value-based prices for the blood test. We assumed that all patients would follow the recommended diagnostic pathway, and real-world experience might reveal drop-outs, which would decrease the value of the blood test. Similarly, the performance of blood tests has mostly been measured under controlled conditions and their accuracy may vary under real-world conditions, which would affect the tests’ economic value, as the sensitivity analysis shows. Our estimate of the value-based price uses cost per patient diagnosed with early-stage AD, and future research should analyze that price using different denominators, such as tested or adjudicated patient, i.e., cases with a definite diagnosis irrespective of etiology.

## 5 Conclusions

Our findings suggest substantial cost savings from incorporating a blood test for AD pathology into the diagnostic pathway. Future research should validate these model-based projections using real-world data and explore the use of denominators other than confirmed cases with early-stage AD on value-based prices. Equally, future research should analyze the additional value that blood tests could generate by reducing bottlenecks in the diagnostic pathway, enabling patients to receive a timelier diagnosis and earlier access to disease modifying therapies.

## CRediT authorship contribution statement

**Soeren Mattke:** Writing – original draft, Project administration, Methodology, Investigation, Funding acquisition, Formal analysis, Data curation, Conceptualization. **Jiahe Chen:** Writing – review & editing, Software, Methodology, Formal analysis. **Mark Hanson:** Writing – review & editing, Methodology, Formal analysis, Conceptualization. **Kim G. Johnson:** Writing – review & editing, Methodology. **Cara Leahy:** Writing – review & editing, Data curation. **David A. Merrill:** Writing – review & editing, Data curation. **Victoria Shada:** Writing – review & editing, Data curation. **Jorge G. Ruiz:** Writing – review & editing, Data curation.

## Disclosures

The work was funding by a contract from C2N to the University of Southern California. The sponsor provided comments on an earlier draft of the manuscript but the authors has full control over the analysis, interpretation of the findings, final draft of the manuscript and decision to submit.

Outside of the submitted work USC has research agreements, on which Dr. Mattke is PI, with Biogen, Eli Lilly, Eisai and Roche/Genentech. Dr. Mattke serves on the board of directors of Senscio Systems and the scientific advisory boards of ALZpath and Boston Millennia Partners. He has received consulting and/or speaker fees from Biogen, C2N Diagnostics, Eisai, Eli Lilly, Novartis, Novo Nordisk, and Genentech/Roche.

Dr. Johnson is the primary investigator of Eisai Inc. AHEAD 3-45 clinical trial on lecanemab therapy for cognitively normal participants, the primary investigator of ALZ-NET at Duke, the primary investigator of LEXEO Therapeutics gene therapy trial, a speaker for Eisai at the 2024 Alzheimer's Association International (AAIC) annual meeting, a consultant with University of Southern California and a Lilly Preclinical Diagnosis Advisory Board member. Dr. Leahy served on advisory boards for Lilly, Eisai and Biogen in the past, and is currently in the speakers’ bureau for Lilly and Eisai as well as a scientific advisor for Neurogen. The other authors report no conflicts.

## Appendix Supplementary materials

Image, application 1
