# Supplementary material for: Estimation of the value-based price of a blood test for Alzheimer’s disease pathology in primary and specialty care in the U.S
Source: J Prev Alzheimers Dis. 2025 Jun 9;12(7):100219. doi: 10.1016/j.tjpad.2025.100219 (PMC12321617; doi:10.1016/j.tjpad.2025.100219)
Supplement: Supplementary file 1 [file mmc1.docx]

# Technical Appendix

## Model input parameters

|  | Value | Reference |
| --- | --- | --- |
| Initial prevalence | | |
| Cognitively normal | 80% | (1) |
| MCI | 14.6% | (1) |
| Dementia | 6.4% | (2, 3) |
| Proportion of MCI due to AD 65-69 | 45% | (4) |
| Proportion of MCI due to AD 70-74 | 51% |  |
| Proportion of MCI due to AD 75-79 | 55% |  |
| Proportion of MCI due to AD 80-84 | 64% |  |
| Proportion of MCI due to AD 85+ | 70% |  |
| Hazard ratio for excess mortality | | |
| MCI | 1.43 | (5) |
| Dementia | 3.26 | (6, 7) |
| Annual transition probability | | |
| Cognitively normal to MCI | 0.030 | (8) |
| MCI to dementia | 0.065 | (9) |
| Initial and confirmatory tests | | |
| MMSE – Sensitivity | 0.82 | (10) |
| MMSE – Specificity | 0.73 |  |
| Blood biomarker test – Sensitivity | 0.88 | (11) |
| Blood biomarker test – Specificity | 0.89 |  |
| Confirmatory cognitive testing – Sensitivity | 0.95 | Assumption |
| Confirmatory cognitive testing – Specificity | 0.95 | Assumption |
| Confirmatory testing with CSF – Sensitivity | 0.91 | (12) |
| Confirmatory testing with CSF – Specificity | 0.89 |  |
| Confirmatory testing with PET – Sensitivity | 0.92 | (13) |
| Confirmatory testing with PET – Specificity | 0.95 |  |
| Proportion of patients receiving amyloid PET scan | 75% | Assumption |
| Proportion of patients receiving CSF testing | 25% | Assumption |

## References

1. Petersen RC, Lopez O, Armstrong MJ, et al. Practice guideline update summary: Mild cognitive impairment: Report of the Guideline Development, Dissemination, and Implementation Subcommittee of the American Academy of Neurology. Neurology. 2018;90(3):126-135. 10.1212/WNL.0000000000004826.

2. Hebert LE, Weuve J, Scherr PA and Evans DA. Alzheimer disease in the United States (2010-2050) estimated using the 2010 census. Neurology. 2013;80(19):1778-83. 10.1212/WNL.0b013e31828726f5.

3. Aneshensel CS, Pearlin LI, Levy-Storms L and Schuler RH. The transition from home to nursing home mortality among people with dementia. J Gerontol B Psychol Sci Soc Sci. 2000;55(3):S152-62. 10.1093/geronb/55.3.s152.

4. Gustavsson A, Norton N, Fast T, et al. Global estimates on the number of persons across the Alzheimer's disease continuum. Alzheimer's &amp; Dementia. 2022. 10.1002/alz.12694.

5. Vassilaki M, Cha RH, Aakre JA, et al. Mortality in mild cognitive impairment varies by subtype, sex, and lifestyle factors: the Mayo Clinic Study of Aging. J Alzheimers Dis. 2015;45(4):1237-45. 10.3233/JAD-143078.

6. Neumann PJ, Araki SS, Arcelus A, et al. Measuring Alzheimer's disease progression with transition probabilities: estimates from CERAD. Neurology. 2001;57(6):957-64. 10.1212/wnl.57.6.957.

7. Murphy SL, Xu J, Kochanek KD and Arias E. Mortality in the United States, 2017. NCHS Data Brief. 2018(328):1-8.

8. Yesavage JA, O’Hara R, Kraemer H, et al. Modeling the prevalence and incidence of Alzheimer’s disease and mild cognitive impairment. Journal of Psychiatric Research. 2002;36(5):281-286. 10.1016/s0022-3956(02)00020-1.

9. Mitchell AJ and Shiri-Feshki M. Rate of progression of mild cognitive impairment to dementia - meta-analysis of 41 robust inception cohort studies. Acta Psychiatrica Scandinavica. 2009;119(4):252-265. 10.1111/j.1600-0447.2008.01326.x.

10. Roalf DR, Moberg PJ, Xie SX, et al. Comparative accuracies of two common screening instruments for classification of Alzheimer's disease, mild cognitive impairment, and healthy aging. Alzheimers Dement. 2013;9(5):529-37. 10.1016/j.jalz.2012.10.001.

11. Meyer MR, Kirmess KM, Eastwood S, et al. Clinical validation of the PrecivityAD2 blood test: A mass spectrometry‐based test with algorithm combining %p‐tau217 and Aβ42/40 ratio to identify presence of brain amyloid. Alzheimer's &amp; Dementia. 2024;20(5):3179-3192. 10.1002/alz.13764.

12. Hansson O, Seibyl J, Stomrud E, et al. CSF biomarkers of Alzheimer's disease concord with amyloid-beta PET and predict clinical progression: A study of fully automated immunoassays in BioFINDER and ADNI cohorts. Alzheimers Dement. 2018;14(11):1470-1481. 10.1016/j.jalz.2018.01.010.

13. Clark CM, Pontecorvo MJ, Beach TG, et al. Cerebral PET with florbetapir compared with neuropathology at autopsy for detection of neuritic amyloid-beta plaques: a prospective cohort study. Lancet Neurol. 2012;11(8):669-78. 10.1016/S1474-4422(12)70142-4.
